# Effect of preterm birth on growth and blood pressure in adulthood in the Pelotas 1993 cohort

**DOI:** 10.1093/ije/dyad084

**Published:** 2023-06-24

**Authors:** Winok Lapidaire, Alvaro Proaño, Cauane Blumenberg, Christian Loret de Mola, Carlos A Delgado, Darwin del Castillo, Fernando C Wehrmeister, Helen Gonçalves, Robert H Gilman, Richard A Oberhelman, Adam J Lewandowski, Jonathan C K Wells, J Jaime Miranda

**Affiliations:** Oxford Cardiovascular Clinical Research Facility, Division of Cardiovascular Medicine, Radcliffe Department of Medicine, University of Oxford, Oxford, UK; Department of Pediatrics, Tulane University School of Medicine, New Orleans, LA, USA; Division of Neonatology, Children's Hospital of Philadelphia, Philadelphia, PA, USA; Post-Graduate Program in Epidemiology, Federal University of Pelotas, Pelotas, Brazil; Causale Consultoria, Pelotas, Brazil; Grupo de Pesquisa e Inovação em Saúde, Programa de Pós-Graduação em Saúde Pública, FURG, Universidade Federal do Rio Grande (FURG), Rio Grande, RS, Brasil; Grupo de Pesquisa e Inovação em Saúde, Programa de Pós-Graduação em Saúde Pública, FURG, Universidade Federal do Rio Grande (FURG), Rio Grande, RS, Brasil; Universidad Científica del Sur, Lima, Peru; Faculty of Medicine, Department of Pediatrics, Universidad Nacional Mayor de San Marcos, Lima, Peru; Neonatal Intensive Care Unit, Instituto Nacional de Salud del Niño, Lima, Peru; CRONICAS Center of Excellence in Chronic Diseases, Universidad Peruana Cayetano Heredia, Lima, Peru; Post-Graduate Program in Epidemiology, Federal University of Pelotas, Pelotas, Brazil; Post-Graduate Program in Epidemiology, Federal University of Pelotas, Pelotas, Brazil; Department of International Health, Bloomberg School of Public Health, Johns Hopkins University, Baltimore, MD, USA; Department of Tropical Medicine, Tulane School of Public Health and Tropical Medicine, New Orleans, LA, USA; Oxford Cardiovascular Clinical Research Facility, Division of Cardiovascular Medicine, Radcliffe Department of Medicine, University of Oxford, Oxford, UK; Population, Policy and Practice Research and Teaching Department, UCL Great Ormond Street Institute of Child Health, London, UK; CRONICAS Center of Excellence in Chronic Diseases, Universidad Peruana Cayetano Heredia, Lima, Peru; School of Medicine, Universidad Peruana Cayetano Heredia, Lima, Peru; George Institute for Global Health, UNSW, Sydney, NSW, Australia

**Keywords:** Preterm birth, blood pressure, low- and middle-income countries, growth

## Abstract

**Background:**

Preterm birth has been associated with increased risk of hypertension and cardiovascular disease later in adulthood, attributed to cardiovascular and metabolic alterations in early life. However, there is paucity of evidence from low- and middle-income countries (LMICs).

**Methods:**

We investigated the differences between preterm (<37 weeks gestational age) and term-born individuals in birth length and weight as well as adult (18 and 20 years) height, weight and blood pressure in the Brazilian 1993 Pelotas birth cohort using linear regressions. Analyses were adjusted for the maternal weight at the beginning of pregnancy and maternal education and family income at childbirth. Additional models were adjusted for body mass index (BMI) and birthweight. Separate analyses were run for males and females. The complete sample was analysed with an interaction term for sex.

**Results:**

Of the 3585 babies included at birth, 3010 were followed up in adulthood at 22 years. Preterm participants had lower length and weight at birth. This difference remained for male participants in adulthood, but female participants were no shorter than their term counterparts by 18 years of age. At 22 years, females born preterm had lower blood pressures (systolic blood pressure −1.00 mmHg, 95%CI −2.7, 0.7 mmHg; diastolic blood pressure −1.1 mmHg, 95%CI −2.4, 0.3 mmHg) than females born at term. These differences were not found in male participants.

**Conclusions:**

In this Brazilian cohort we found contrasting results regarding the association of preterm birth with blood pressure in young adulthood, which may be unique to an LMIC.

Key Messages

- Preterm-born females in a low- and middle-income setting have lower blood pressures in adulthood compared with term-born females.
- Preterm-born males in a low- and middle-income setting were shorter and weighed less in infancy and in adulthood than their term counterparts.
- Preterm-born females in a low- and middle-income setting had lower length at birth, but by 18 years of age they were no shorter than term female controls.

## Introduction

Prematurity is an important risk factor for infant mortality, both in high-income countries (HIC) and low- and middle-income countries (LMICs), with lower gestational age being associated with higher mortality.^1–3^ The global prevalence of prematurity ranges between 5% and 18%, with lower estimates observed in HICs and higher figures in LMICs.^4^^,^^5^ Besides infant mortality, prematurity has also been associated with increased morbidity into adulthood, based on large birth registry studies conducted in HICs.^6^ Adults born preterm have shown to be at greater risk of cardiovascular disease, including early onset hypertension,^7^ heart failure,^8^ ischaemic heart disease,^9^ type 2 diabetes^10^ and early cardiovascular-related mortality.^11^

The link between prematurity and disease later in life is part of the developmental origins of health and disease theory, where disease traits in adult life can trace back to infancy, birth and/or pregnancy periods. There is a body of evidence for a variety of outcomes across LMICs and HICs,^12–14^ but the scientific literature on prematurity is predominantly based on HICs. Observational studies from HICs have demonstrated unique alterations in the cardiometabolic systems of adults born preterm which may make them more susceptible to developing cardiovascular disease.^15^ Evidence from HICs has shown that there is an association between prematurity and blood pressure, fat distribution and height attained by adulthood. Current knowledge shows an association between adults born preterm with higher blood pressure, and two recent meta-analyses showed that adolescents and adults born preterm have higher levels of blood pressure compared with their term-born peers,^16^^,^^17^ particularly in women.^17^ Additionally, preterm children are associated with lower absolute fat mass with a more central fat distribution, which is an independent cardiometabolic risk factor.^18^ Similarly, adults born preterm have shown to have an increased visceral fat distribution^19^ and to be shorter than their term-born peers.^20^^,^^21^

However, LMICs pose a different environment, with six times the prevalence of intrauterine growth restriction of the fetus when compared with HIC, and there is new evidence that questions the prior hypothesis of early origins of cardiovascular disease in the LMICs.^14^^,^^22^ This led us to investigate further how prematurity plays a role in cardiovascular disease in an LMIC. We sought to investigate blood pressure and body composition of adults who were born preterm in an LMIC. Based on the aforementioned studies performed in HICs, our null hypothesis is that adults born preterm in an LMIC would have higher blood pressure and body mass index (BMI) and that they would be shorter compared with adults born at term.

## Methods

### Study design and setting

Brazil is considered one of the top 10 countries identified as having the highest prevalence of prematurity, with over 3 million preterm births in 2010.^5^ The 1993 Pelotas cohort has detailed birth history data with multiple follow-up points into young adulthood, affording an opportunity to assess the impact of preterm birth on blood pressure and body composition at different ages. The 1993 Pelotas birth cohort is a population-based, longitudinal study that invited all mothers who lived in urban areas of the city and gave birth in maternity hospitals in the 1993 calendar year in Pelotas, Brazil. Pelotas is a medium-sized city in Brazil with approximately 340 000 inhabitants, in which 92% of the population lives in urban areas, with a human development index of 0.739 in 2010.^23^ In 1993, 5265 live births were eligible and, 5249 mothers agreed to participate and were interviewed soon after giving birth. This study relies on data from follow-ups in the perinatal period and at 18 and 22 years of age. For the follow-ups at 18 and 22 years of age , all cohort members were sought and invited to participate, reaching follow-up rates equal to 81.4% and 76.3%, respectively. Further details on study methodology have been published elsewhere.^24–26^ Ethical approval was obtained from the Federal University of Pelotas Medical School Ethics Committee.

Preterm birth was defined as at gestational age <37 weeks. Birthweight Z-scores were calculated based on Intergrowth reference data.^27^ Intergrowth data include the sex of the newborn, to classify anthropometry data. Those with a Z-score below −1.28 were classified as small-for-gestational-age and those with a Z-score above 1.28 were defined as large-for-gestational-age. These Z-scores correspond to the 10th percentile and 90th percentile, respectively. We excluded participants who had missing gestational age or birthweight or those not followed up after birth. Additionally, we excluded unrealistic gestational ages (<18 weeks or >45 weeks, *n* = 577) or unrealistic birthweights (Z-scores <−3 or >3, *n* = 314).

### Background variables

Maternal information was collected through a perinatal interview. Family income at the time of birth was assessed in multiples of minimum wage per month (in 1993, 1 minimum wage = US$ 31.4 per month). Maternal smoking and alcohol consumption data during pregnancy were collected retrospectively at birth. Maternal age was recorded in relation to the date of the child’s birth.

### Exposure

All preterm babies were considered exposed, and prematurity was classified according to estimated gestational age (<37 weeks). Estimation of gestational age was based on the last menstrual period. When information from the last menstrual period was unknown or not reliable, the clinical maturity estimate based on the Dubowitz method was used.^28^

### Outcomes

Outcomes included the following. Catch-up growth defined birthweight and length at birth and height at 18 years and 22 years using traditional anthropometry. Birthweight was obtained based on hospital registries. Standing height at 18 and 22 years was measured at the research clinic, with a fixed stadiometer with 0.1 cm precision. Weight in the birth cohort follow-up at 22 years was measured using air-displacement plethysmography (BodPod^®^ Gold Standard, USA).^26^ BMI was calculated by dividing weight (kg) by height (m) squared. Systolic and diastolic blood pressures at 22 years were measured after a 10-min rest, using an automated sphygmomanometer (model HEM-705CP INT, OMRON, Beijing) with a margin of error of 1 mmHg.^26^

### Statistical analysis

Statistical analyses were performed in R (version 4.5.0).^29^ Descriptive statistics are presented as absolute and relative frequencies. Categorical data are presented as percentages and quantitative statistics for non-normal data are presented as median and the interquartile range (IQR).

Analyses were adjusted for the following maternal factors: maternal weight at the beginning of pregnancy, maternal education at childbirth and family income at childbirth. Linear regressions with blood pressure and growth parameters as dependent variables, preterm birth status (preterm/term) as independent variables, and maternal factors as the covariates were run to assess the effect of preterm birth on height, body composition, growth and blood pressure in adulthood. Participants with missing data were excluded from the analysis.

Three models were run for each outcome variable.^30^ Model 1 was adjusted for maternal factors, Model 2 was adjusted for maternal factors and BMI and Model 3 was adjusted for maternal factors, BMI and birthweight.

As sexual dimorphism in adult size, body composition and blood pressure are well established and have their origins in patterns of growth, development and tissue accretion in early life,^31–33^ we analysed the two sexes separately. Separate analyses were run for males and females. To assess whether the effect of preterm birth on outcomes differed between males and females, an additional model was created for each regression analysis with males and females combined with an interaction term. We focus our discussion on the findings on the magnitude of effect, and provide confidence intervals.

## Results

At baseline, 3207 term (51% females) and 378 preterm (52% females) babies were included in this study. At the 22-year follow-up, data from 2696 adults born at term (52% females) and 314 adults born preterm (56% females) were analysed. Of these adults, none were born extremely preterm (<28 weeks) and 18 were born very preterm (28–32 weeks). Table 1 shows the participant characteristics of the preterm and term groups. On average, maternal education of preterm babies was 1 year lower than term babies, but both groups had low maternal education. Preterm and male term babies belonged to households with lower family income than female term babies. Supplementary Table S1 (available at *IJE* online) shows the participant characteristics of those who were not included in the analyses due to loss to follow-up at 18 and 22 years.

**Table 1.** Participant characteristics at baseline

	Preterm		Term	
Characteristic	Female	Male	Female	Male
Participants at baseline, *n* (%)	196 (5%)	182 (5%)	1629 (45%)	1578 (44%)
Participants at age 18 years, *n* (%)	176 (5%)	167 (5%)	1481 (45%)	1495 (45%)
Participants at age 22 years, *n* (%)	176 (6%)	138 (5%)	1399 (47%)	1297 (43%)
Maternal smoking during pregnancy, *n* (%)	78 (2%)	65 (2%)	533 (15%)	482 (13%)
Maternal alcohol intake during pregnancy, *n* (%)	17 (<1%)	5 (<1%)	84 (2%)	77 (2%)
Family income at baseline (multiple of minimum wage), median (IQR)	2.4 (1.4, 4)	2.5 (1.5, 4.5)	3 (1.5, 5.0)	2.6 (1.5, 4.8)
Maternal education (years), median (IQR)	5 (4, 8)	6 (4, 8)	6 (5, 9)	6 (5, 9)
Maternal age (years), median (IQR)	26 (21, 32)	26 (20, 31)	26 (21, 31)	26 (21, 31)
Birth length (cm), median (IQR)	47 (45, 49)	48 (46, 50)	49 (48, 50)	50 (48, 51)
Birthweight (g), median (IQR)	2625 (2278, 3000)	2820 (2405, 3218)	3170 (2880, 3470)	3300 (3000, 3600)
Small for gestational age, *n* (%)	17 (1%)	10 (<1%)	288 (8%)	261 (7%)

IQR represents interquartile range.

Table 2 shows that male preterm participants had lower weight and length at birth as compared with their term-born counterparts, and continued to have a lower height at ages 18 and 22 years. Preterm-born female participants, although having lower length and weight at birth, were on average no shorter than term-born females at 18 and 22 years. Females born preterm grew 2.6 cm more between birth and age 22 years than females born at term, and such difference was not observed among males. There was evidence of an interaction between sex and preterm status: male preterms grew shorter relative to male terms, when compared with female preterms relative to term females (1.91 cm, p = 0.02). There was no evidence of an interaction effect between sex and preterm status on weight or length at birth or on weight or BMI at age 22 years. Supplementary Table S2 (available at *IJE* online) shows the differences between preterm and term groups in growth parameters for each gestational age category (small, appropriate and large for gestational age).

**Table 2.** Differences in growth parameters between preterm and term groups, in male and female participants

	Female		Male	
Growth parameter	*n*	Preterm relative to term^a^	*n*	Preterm relative to term^a^
Length at birth (cm)	1809	**–2.1 (–2.5, –1.8)**	1750	**–1.8 (–2.1, –1.5)**
Weight at birth (kg)	1825	**–0.5 (–0.6, –0.5)**	1760	**–0.5 (–0.5, –0.4)**
Height at age 18 years (cm)	1657	0.0 (–1.0, 1.0)	1662	**–1.2 (–2.3, –0.1)**
Height at age 22 years (cm)	1575	0.5 (–0.5, 1.5)	1435	**–1.3 (–2.5, –0.1)**
Growth from birth to age 22 years (cm)	1562	**2.6 (1.6, 3.6)**	1426	0.4 (–0.7, 1.6)
Weight at age 22 years (kg)	1575	0.9 (–1.4, 3.3)	1435	–1.7 (–4.5, 1.0)
BMI at age 22 years (kg/m^2^)	1565	0.3 (–0.6, 1.2)	1426	–0.2 (–1.0, 0.6)

BMI, body mass index.

a Coefficient for preterm status (95% confidence interval). Significant differences at *P *<0.05 are shown in bold.

**Figure 1.**
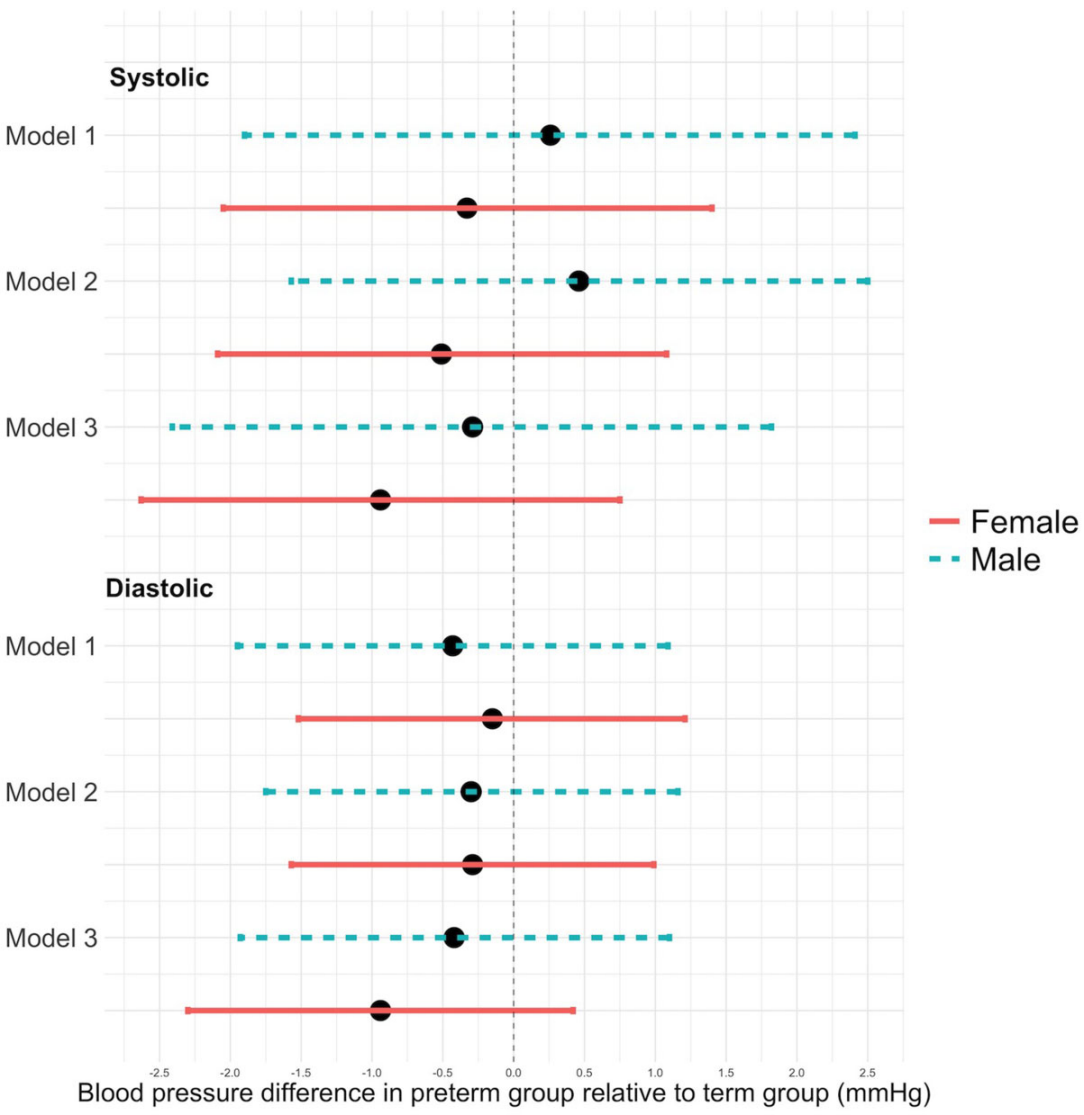
Forest plot of systolic and diastolic blood pressure differences between preterm and term groups in males and females. Model 1 is adjusted for maternal factors (maternal weight at the beginning of pregnancy, maternal education at child birth and family income at child birth), Model 2 is adjusted for maternal factors and body mass index at 22 years, and Model 3 is adjusted for maternal factors, body mass index at 22 years and birthweight

Figure 1 shows differences in blood pressure in 22-year-old males and females born preterm compared with those born at term. In Models 1 and 2, all blood pressure contrasts between preterm and term individuals were <1 mmHg, and with one exception all were <0.5 mmHg. However in Model 3, preterm women showed lower systolic blood pressure (SBP: −0.94 mmHg, 95% CI −2.6, 0.8 mm Hg) and lower diastolic blood pressure (DBP: −0.94 mmHg, 95% CI −2.3, 0.4 mm Hg), and these differences were not observed in men to the same extent (SBP: −0.3 95% CI −2.4, 1.8 and DBP: −0.4 mmHg 95% CI −1.9, 1.1).

## Discussion

Leveraging from a longitudinal Brazilian birth cohort study with follow-up data into early adulthood, this study found that there was no significant association between preterm birth and blood pressure in young adulthood in this LMIC. Preterm individuals were shorter than their term counterparts in early life, yet at 22 years of age, women had caught up to their female counterparts born at term whereas this was not observed in males. Contrasting with studies from HICs,^7^^,^^34–38^ we did not find evidence of higher blood pressure in preterm babies compared with those born at term, rather the opposite was true for preterm females, who demonstrated lower blood pressure than their counterparts born at term when adjusting for birthweight in addition to maternal factors and BMI.^7^^,^^34–38^ To our knowledge, this is the first longitudinal study into adulthood evaluating the patterns and levels of blood pressure by preterm status in Latin America.

These findings may be affected by survivor bias in the cohort. Our cohort had a smaller number of preterm infants with lower gestational ages and lower birthweight, which could be due to higher mortality in the setting studied. Indeed, our Supplementary Figure S2 shows that those who died between birth and 22 years were more likely to have a lower birthweight. Since there are no surviving small-for-gestational age infants born below 32 weeks of gestation, our preterm sample was biased towards those who were appropriate and large for gestational age (see Supplementary Figure S1). The survivor bias is stronger in LMICs, contributing to the literature on long-term effects of prematurity on cardiovascular-related outcomes, given the vast difference in neonatal mortality in LMICs when compared with HICs.^39^^,^^40^ This is similar to the large Nordic registry that found an association between size for gestational age and mortality in the first 18 years of life, particularly when small-for-gestational-age infants were born preterm.^41^ Therefore, the difference in blood pressure between preterm and term-born groups might be smaller in this Brazilian cohort than has previously been reported in HIC populations. If a large number of people with lower gestational age and lower birthweight preterm babies would have survived to adulthood, we would expect a higher mean blood pressure for the preterm population. Similarly, catch-up growth might have been reduced in females of lower gestational age and/or birthweight.

The male disadvantage hypothesis suggests that males are more sensitive to adverse factors during development than females.^42–44^ The sex disparity in the effects of preterm birth is likely multifactorial and may include hormonal, genetic and immunological differences. Males are more vulnerable to infectious diseases,^45^ as well as to the exposure to certain hormones that increases the incidence of wasting and stunting in infancy.^46^ A review of three studies of birth cohorts in Pelotas, Brazil (including data from the 1993 cohort analysed in this study), revealed preterm birth was associated with body composition at 6, 18 and 30 years of age in males only.^47^ Preterm birth was associated with decreased body fat and fat-free mass in childhood but increased fat mass in adulthood.

Systolic and diastolic blood pressure was lower in women born preterm compared with those born at term, whereas in men the difference was negligible. Again, these effects observed in women are in the opposite direction to that reported in studies from high-income settings.^7^^,^^34–38^ One possible factor contributing to this sex difference could be the greater catch-up in height in women compared with men. In both sexes, those born preterm were around 2 cm shorter at birth in absolute terms, compared with those born at term. However, whereas this difference had disappeared in women at 22 years, it remained in men. However, adjusting for adult height and body composition made little difference to the blood pressure difference between preterm and term individuals in either sex, suggesting that this catch-up, although potentially important for health, may not have been an important causal factor in the blood pressure contrasts.

Importantly, the lower blood pressure of preterm women only became evident in Model 3, which included additional adjustment for birthweight. In many studies, a lower birthweight has been associated with a higher blood pressure.^16^^,^^17^^,^^48^ This may again indicate effects of survivor bias, such that in this population, preterm birth status in women acts as a reliable marker of ‘above average fetal growth’, resulting in lower blood pressure when adjusted for maternal factors, BMI at 22 years and birthweight. Another hypothesis is known as the ‘birthweight paradox’,^30^ which in this context would propose that the association of birthweight and blood pressure may change when stratified on another exposure, i.e. preterm birth, female sex or both. Whereas it is possible that individuals born preterm have a different biological association between birthweight and blood pressure compared with those born at term, we suggest that it is more likely that our results are generated by our sample missing preterm small-for-gestational age (SGA) individuals. By having lost the individuals with the lowest metabolic capacity, the group of preterms appears better able to tolerate their adult metabolic load. Why this beneficial effect is stronger in females is less clear. Possibly the effect of having lower birthweight due to being female does introduce a collider effect; this would be worth studying in the future.

There is evidence that the risk of non-communicable diseases such as cardiovascular diseases, hypertension and type 2 diabetes, is shaped both by adult traits and by early developmental experience. Maternal exposures such as malnutrition and psychosocial stress are associated with poor growth of the offspring *in utero*, which has long-term detrimental effects on the capacity for metabolic homeostasis.^49^ A recent review demonstrated the long-lasting effects of prematurity on cardiovascular structure and function, in both the left and the right heart, with increased systolic and diastolic blood pressures.^50^ Additionally, the catch-up weight that preterm individuals need to gain can predispose them to a fast rate of fat accumulation which could be a risk factor for chronic diseases, including metabolic and cardiac.^6^^,^^51^

Regarding hypertension, two meta-analyses reported an increase in SBP in those born preterm compared with term.^16^^,^^48^ The results showed high consistency, with preterm individuals showing higher BP in all 20 SBP studies and in 95% of the studies that evaluated DBP. Yet these meta-analyses included only one study conducted in an LMIC. New evidence is starting to show differences in cardiovascular outcomes for those born preterm in an LMIC. A study from the 1982 Pelotas Cohort also showed no association between preterm birth and higher blood pressure but did find an association when comparing hypertension and those born small for gestational age in a smaller population (total *n* = 1076 adolescents) of participants who were up to 15 years old.^52^ The current study evaluates a larger sample size of individuals up to young adulthood (*n* = 3010 young adults). The Birth to Twenty Cohort, an African cohort from Soweto, Johannesburg, with 1540 young adults, also did not find any association between prematurity and hypertension in young adults when comparing them at age 23 years, which is in line with our results.^53^ Being the largest study to date examining this association in an LMIC, our study helps highlight that cardiovascular outcomes for premature infants might be different from what has been previously reported in HICs.

In our study the reduction in blood pressure in preterm women reached borderline statistical significance, yet the magnitude of effect is meaningful in public health terms. For example, a reduction of salt intake of 3 g/d reduces SBP by 3.2 to 3.6 mm Hg among individuals with hypertension, and by 1.8 mm Hg per day in normotensives.^54^ Slightly larger effects of physical activity on blood pressure have been reported. Population-based studies of modest reductions in levels of blood pressure (<2 mm Hg) are accompanied by substantial reductions on the incidence of hypertension.^55^

The results observed in the opposite direction as expected, i.e. lower blood pressure among adults born preterm rather than high blood pressure, demonstrate the unique contributions of studies conducted in LMICs. This aligns with the results from the 1982 Pelotas Cohort as well as the Birth to Twenty Cohort from Johannesburg, showing no real difference in adult blood pressure in those who were born preterm.^52^^,^^53^ Further studies are needed, focusing on the long-term impacts of prematurity in LMICs while taking into consideration the survival bias effect. In addition, future studies could use 24-h blood pressure monitoring systems and examine whether end organ changes in the heart, brain, eye and kidney are associated with preterm birth in this population, as has been reported in high-income settings.^56–59^

There are some strengths of this study. In terms of representativeness of the data, the 1993 Pelotas birth cohort included 99.7% of all births happening in the city in that year. The follow-up rates were also high, ranging from 76.3% to 81.4%, at least within longitudinal studies.^26^ In addition, the data collected in different cohort follow-ups adhered to a standardized protocol always performed by trained personnel. A few limitations should also be noted. Whereas sample size may have affected the power to reach statistical significance in some of the observations presented, the effect sizes and directionality of the results are substantially informative, particularly considering the paucity of long-term studies of prematurity in LMICs. Gestational age assessment was based on the last menstrual period, which has been shown to be an estimate to the gold standard (ultrasound) in resource-limited settings.^60^ However there is still a difference between both, given that the last menstrual period will be affected by recall bias which can contribute to definition problems such as determining a preterm birth when it is not.^61^ Assuming that the preterm newborn has to recover the lost weight in the shortest possible time, it can lead to an excessive accumulation of fat that increases the risk of metabolic and cardiovascular problems in adult life.^51^ However, there is no growth standard for preterm infants and fetal growth is often used as the reference parameter. The best standard at this time is the INTERGROWTH reference data, which were used in this analysis.^27^ If practically feasible, ambulatory blood pressure monitoring could be used in future studies as this method has a stronger association with cardiovascular disease risk compared with the office-based measurement available in this study.^62–65^ The ascertainment of cardiovascular outcomes in early adults is challenging, as many of these conditions will have onset later into adulthood. Nevertheless, blood pressure is a good marker of cardiovascular health and a major contributor to the global burden of disease.^66^ Additionally, in our cohort we were not able to adjust for gestational diabetes or hypertension in our analysis due to the lack of reliable data.

As the prevalence of prematurity survivors increases, it may be necessary to explore whether the current risks of yesterday's preterm infants are indicative of the future risks of tomorrow's preterm infants in LMICs.^67^ Understanding the medium- or long-term consequences of prematurity for hypertension in LMICs is vital, as most preterm births^5^ and cardiovascular disease^68^ occur in LMICs. This study starts to address the paucity of research on prematurity in LMICs. The results indicate that data from HICs cannot be directly translated to LMIC populations, which has important implications for the interpretation and global application of medical research more generally. Future studies should collect data prospectively and use advanced (imaging) equipment to assess cardiovascular alterations in more detail in multiple LMICs.

## Conclusion

In conclusion, our analyses add to growing literature indicating that in LMICs, the association of preterm birth with blood pressure in young adulthood may be different from that reported in HIC. This may relate to differential rates of survival of the smallest preterm infants, contributing to a healthy survivor effect. The long-term implications of preterm birth for health in LMICs needs further research, as adverse effects may become evident at older ages in the life course, and this potential health burden has yet to be characterized.

## Ethics approval

Ethical approval was obtained from the Federal University of Pelotas Medical School Ethics Committee (register number 05/2011 and 1.250.366).

## Supplementary Material

dyad084_Supplementary_Data

## Data Availability

This article is based on data from the study ‘Pelotas Birth Cohort, 1993’ conducted by the Postgraduate Program in Epidemiology at the Universidade Federal de Pelotas with the collaboration of the Brazilian Public Health Association (ABRASCO) and patronized, from 2004 to 2013, from the Wellcome Trust. The data underlying this article were provided by the Federal University of Pelotas, Brazil. Data will be shared on request to the corresponding author with permission of the Federal University of Pelotas, Brazil.
