# Supplementary material for: Effect of preterm birth on growth and blood pressure in adulthood in the Pelotas 1993 cohort
Source: Int J Epidemiol. 2023 Jun 24;52(6):1870–7. doi: 10.1093/ije/dyad084 (PMC10749774; doi:10.1093/ije/dyad084)

Supplementary Table S1: Characteristics of participants excluded from analyses

	Preterm	Term
Female n (%)	68 (43%)	458 (49%)
Small for gestational age n (%)	83 (6%)	58 (22%)
Family income - minimum wage Median (IQR)	2.0 (1.0,3.6)	2.7 (1.5,5.0)
Maternal education - years Median (IQR)	5 (3,8)	6 (4,10)
Length birth - cm Median (IQR)	48 (46,50)	49 (48,50)
Weight birth - g Median (IQR)	2850 (2300,3300)	3200 (2890,3505)
Gestational age - weeks Median (IQR)	34.4 (31.6-36)	40.4 (39.1,42.4)
Height 18 yrs - cm Median (IQR)	166 (161,175)	166 (159,173)
Height 22 yrs - cm Median (IQR)	165 (158,172)	167 (158,174)
Growth birth-22 years - cm Median (IQR)	115 (109,121)	117 (111,124)
BMI 22 yrs - Median (IQR)	24.4 (22.2,27.9)	24.7 (21.5,28.2)
SBP 22 yrs - mmHg Median (IQR)	121 (111,129)	123.2 (114,131)
DBP 22 yrs - mmHg Median (IQR)	70 (65,76)	73 (67,79)

Supplementary Table S2: Differences between preterm and term groups on growth parameters for each gestational age category; small (SGA), appropriate (AGA), and large for gestational age (LGA)

	SGA	AGA	LGA
	preterm vs term	preterm vs term	preterm vs term
n preterm, n term	549, 31	306, 123	2352, 228
Length at birth (cm)	-4.6 (-5.4,-3.9)	-2.7 (-2.9,-2.4)	-2.4 (-2.9,-1.9)
Weight at birth (kg)	-0.8 (-0.9,-0.7)	-0.7 (-0.7,-0.6)	-0.8 (-0.9,-0.7)
Height 18 yrs (cm)	0.0 (-3.6,3.5)	-1.4 (-2.6,-0.1)	-3.4 (-5.5,-1.3)
Height 22 yrs (cm)	0.0 (-3.8,3.8)	-1.2 (-2.6,0.2)	-3.7 (-6.0,-1.5)
Weight 22 yrs (kg)	-1.9 (-8.5,4.8)	-0.8 (-3.2,1.6)	-3.9 (-7.9,0.1)
BMI 22 yrs (kg)	-0.7 (-2.8,1.5)	0.2 (-0.6,1.0)	0.2 (-1.5,1.1)

Coefficient for preterm status (95% CI). Significant differences between preterm and term born participants at p<0.05 are presented in bold.

Supplementary Figure S1: Proportion of small (SGA), appropriate (AGA), and large (LGA) for gestational age participants by gestational age

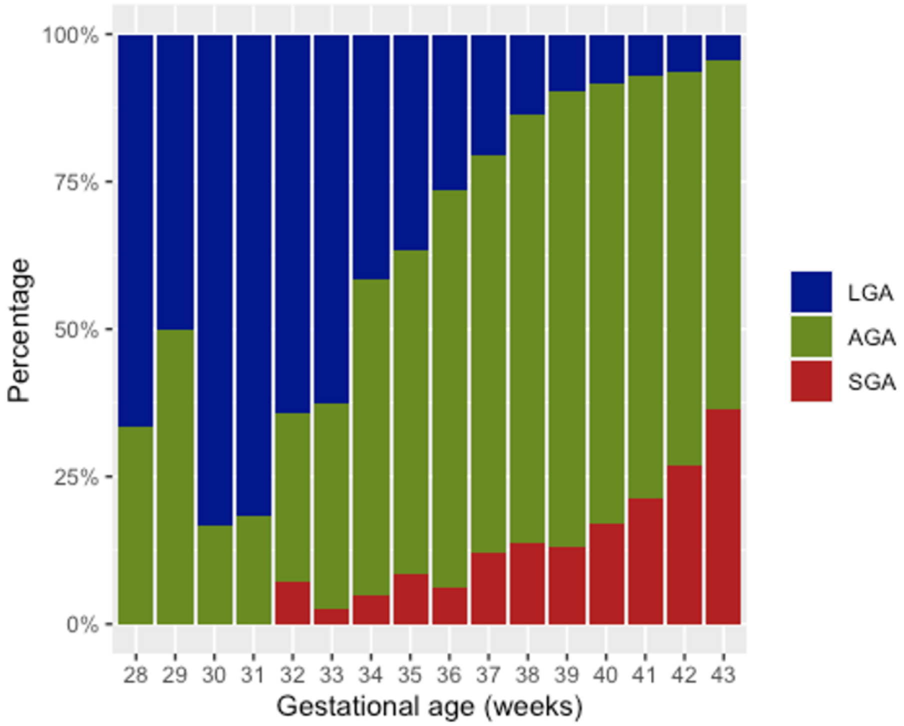

Supplementary Figure S2: Birthweight Z-score (A) and sex (B) of those followed up and those not followed up at age 22 years due to refusal, death or reasons unknown.

A

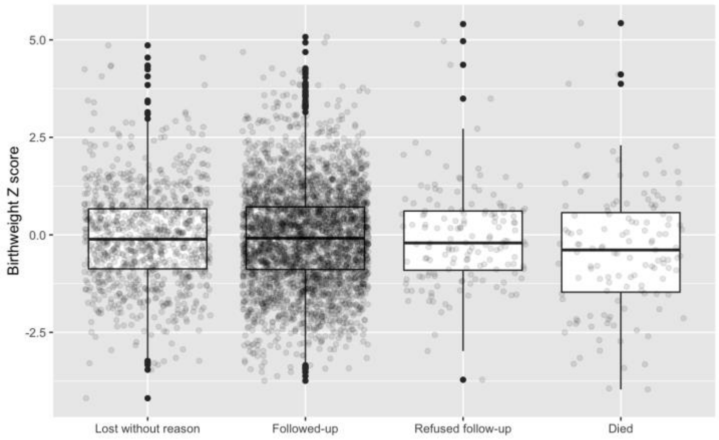

B

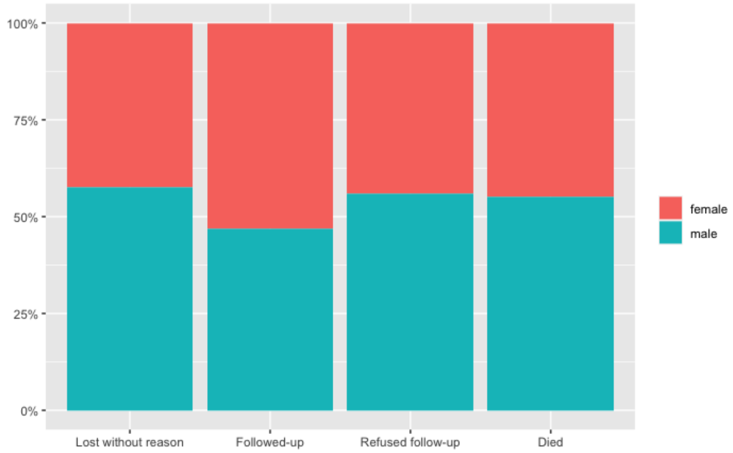

Supplement: dyad084_Supplementary_Data [file dyad084_supplementary_data.pdf]
